# Giant sliding inguinal hernia requiring intraoperative aspiration of fluid: a case report and literature review

**DOI:** 10.1093/jscr/rjab340

**Published:** 2021-08-26

**Authors:** Tyler Davis, Mica Vivens, Lutfi Barghuthi, Hishaam Ismael

**Affiliations:** General Surgery, University of Texas at Tyler, Tyler 75708, USA; General Surgery, University of Texas Medical Branch at Galveston, Galveston, USA; General Surgery, University of Texas at Tyler, Tyler 75708, USA; General Surgery, University of Texas at Tyler, Tyler 75708, USA

## Abstract

Giant inguinal hernias are defined as inguinal hernias that extend below the midpoint of the inner thigh when the patient is in the standing position or an anteroposterior diameter of at least 30 cm or a laterolateral diameter of ~50 cm with non-reducibility for >10 years. This article presents a 39-year-old male with a five-year history of a giant left inguinal hernia that was treated with left inguinal hernia repair with mesh, orchiectomy, complicated scrotoplasty, intraoperative ultrasound and aspiration of 3.9 L of fluid from the hernia sac. Surgical repair of giant inguinal hernias can be challenging and is associated with a variety of complications. Various modalities have been described to assist in hernia reduction including debulking, or, as in this case, aspiration of the hernia sac and a preperitoneal incision. Although the Lichtenstein tension free repair is commonly used, no standard approach has been accepted.

## INTRODUCTION

Abdominal wall hernias are extremely common with an estimated prevalence of 1.7% for all ages. With roughly 75% being inguinal in nature, repair of said hernias is one of the most common surgical operations performed. However, if left untreated, simple inguinal hernias can lead to many complications. One of which is the development of a giant inguinal hernia.

This is defined as an inguinal hernia that extends below the midpoint of the inner thigh when the patient is in the standing position or an anteroposterior diameter of at least 30 cm or a laterolateral diameter of ~50 cm with non-reducibility for >10 years. Contents of giant inguinal hernias are usually peritoneal fat and bowel but reports of inclusion of stomach, ovaries, bladder and kidneys have also been noted. Complications are common with giant inguinal hernias and include urinary retention, reduced mobility, pressure sores, social isolation and intestinal incarceration/strangulation.

Problems associated with surgical management of giant inguinal hernias are not uncommon and include loss of domain, abdominal compartment syndrome, cardiorespiratory complications, recurrence and wound breakdown. Though several repair techniques have been suggested in published case reports, no single treatment has been adopted as the standard approach to dealing with this unusual disease.

This article is an examination of a 39-year-old Hispanic male with a rare giant left-sided sliding inguinal. This patient underwent intraoperative ultrasound with the aspiration of hernia, orchiectomy, complicated scrotoplasty and repair with mesh.

## PRESENTATION OF CASE

**Figure 1:**
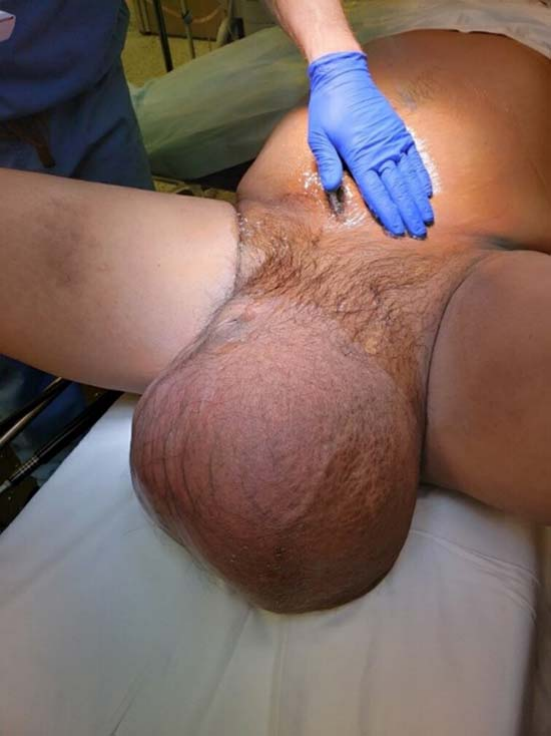
Preoperative gross appearance of giant inguinal hernia.

The patient is a 39-year-old Hispanic male who presented to the surgery clinic with a five-year history of a giant left inguinal hernia. On physical exam, he had a left-sided ‘basketball-sized’ inguinal hernia (shown in Fig. 1). Bowel sounds were auscultated in the scrotum, suggesting a significant portion of bowel contents had been displaced in the scrotum. The patient’s left testicle could not be palpated.

**Figure 2:**
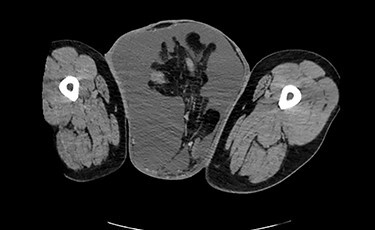
CT (axial view) demonstrating large left inguinal hernia sac containing bowel, fluid and left ureter.

**Figure 3:**
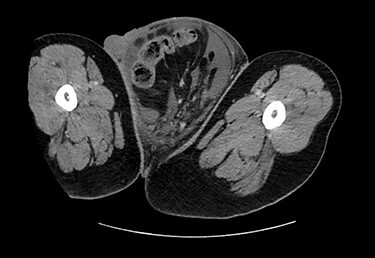
CT (axial view).

Testicular ultrasound revealed a large left inguinal hernia with a fluid-filled hernia sac surrounding one or more segments of the intestine within the scrotum, measuring >15 cm. The left testis could not be identified. The right testis appeared normal on imaging but was displaced superiorly and medially by the large left scrotal hernia sac. The right testis demonstrated color Doppler signals and arterial Doppler waveform. Computed tomography (CT) revealed a large left inguinal hernia sac with bowel, fluid and Left ureter within it associated with left-sided hydronephrosis and severe cortical thickening. Figures 2 and 3 demonstrate pertinent CT images.

**Figure 4:**
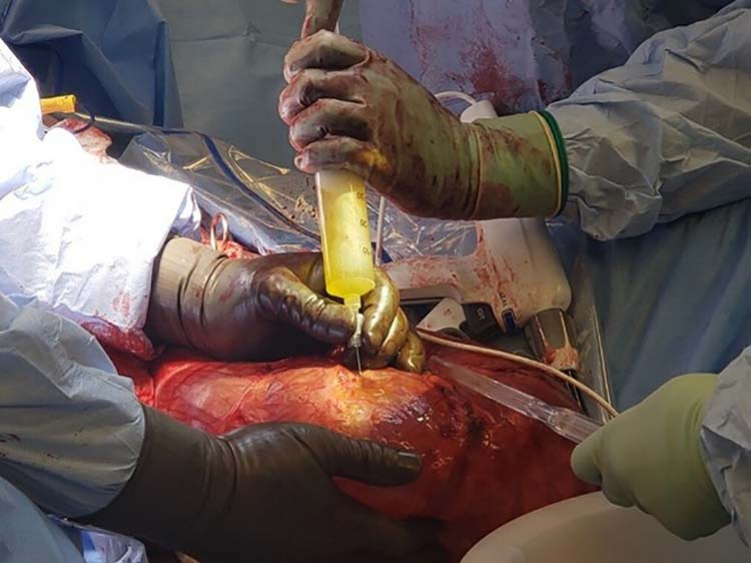
Intraoperative picture demonstrating fluid being drawn from hernia sac.

**Figure 5:**
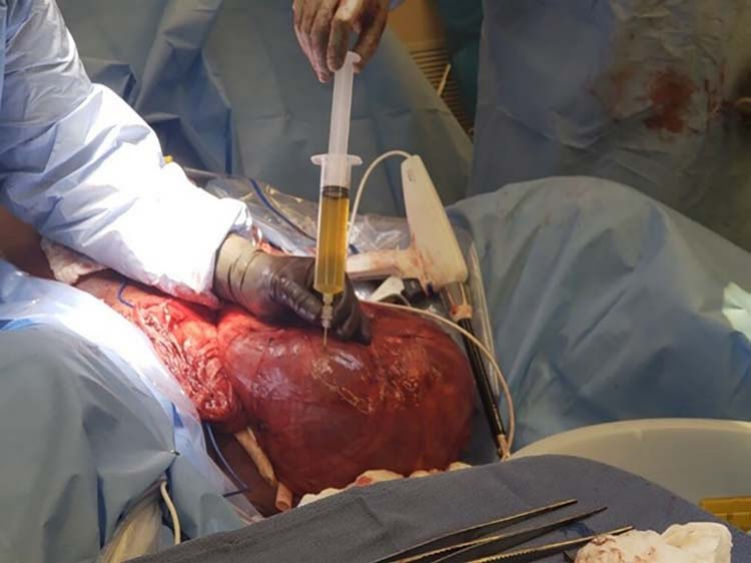
Intraoperative picture of hernia sac after decompression.

**Figure 6:**
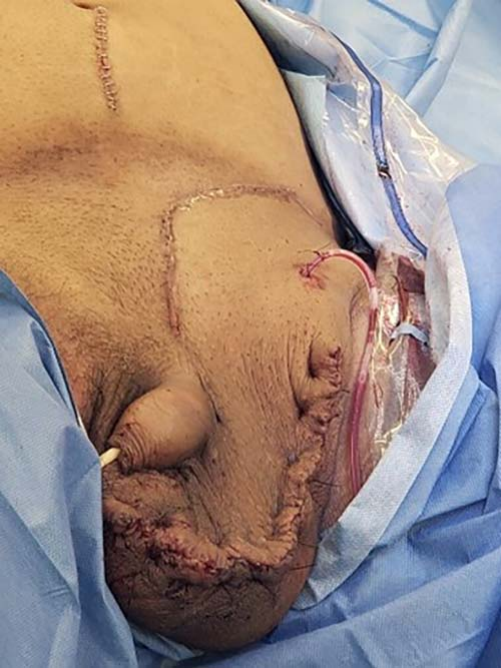
Postoperative closure of hernia.

The patient underwent left inguinal hernia repair with mesh, orchiectomy, complicated scrotoplasty, intraoperative ultrasound and aspiration of 3.9 L of fluid from the hernia sac. General surgery and urology were both present for the operation. A generous left inguinal incision was initially used and eventually extended as a hockey stick down to his scrotum, lateral to the penis. The hernia sac was taken off the left spermatic cord and testicle. The testicle was inspected and appeared atrophic, with severe elongation and splaying of the spermatic cord from prolonged stretching due to the hernia. Thus, a left orchiectomy was performed by urology. Ultrasound-guided aspiration of the hernia fluid was then performed, yielding 3.9 liters of hernia fluid. Figure 4 shows the fluid being drawn from the hernia sac. Figure 5 demonstrates the hernia sac after decompression. The hernia sac was subsequently opened anteriorly revealing the ascending colon and ureter to form a sliding hernia. Half of the hernia sac was removed, whereas the remaining sac was closed with running 2–0 Vicryl suture. After a failed attempt at hernia reduction, an infraumbilical incision was then made. This exploratory laparotomy did not enter the peritoneum. The preperitoneal space was entered and extended to the hernia sac, which allowed the hernia to be pushed distally and pulled superiorly to successfully reduce the hernia. A scrotoplasty was then performed, and the hernia was reinforced using a 6 cm x 8 cm Phasix mesh in a Lichtenstein tension free fashion. The patient tolerated the procedure very well, with an uncomplicated postoperative course and returned to work six-week later. Figure 6 demonstrates the final closure.

**Table 1:** Literature review of 11 cases of giant inguinal hernias

Author/Article	Year	Age	Gender	Presentation/Exam	Workup	Incision/Approach	Debulking required?	If debulked, what was done?	Hernia Repair technique
Trakarnsagna *et al.*: Giant inguinal hernia: Report of a case and reviews of surgical technique	2014	67	M	Right-sided inguinal hernia for past 30 years with symptoms of partial colonic obstruction, weight loss	Barium Enema: ascending colon, cecum, ileum in hernia sac.	Right inguinal transverse incision	Yes	Partial omentectomy	Lichtenstein’s tension free
Karthikeyan *et al.*: Giant inguinoscrotal hernia- report of a rare case with literature review	2014	50	M	Right-sided inguinal hernia with bilateral scrotal swelling for 25 years and buried penis, dilated veins over scrotum and expansile cough impulse	U/S: bowel loops, bilateral hydrocele Intraoperative hernia sac contents: entire greater omentum, small bowel, appendix	Right inguinal transverse incision	Yes	Partial omentectomy, bilateral subtotal excision of sac, right orchidectomy	Lichtenstein’s tension free
Qaja *et al.*: Repair of giant inguinoscrotal hernia with loss of domain	2017	74	M	Left-sided inguinal hernia for past 30 years with dilated veins over scrotum and bowel peristalsing in left scrotum.	CT: entire omentum, small bowel, appendix, large bowel within hernia sac	Left inguinal transverse incision	Yes	Partial Omentectomy, small bowel resection	Modified Lichtestine’s
Wang *et al.*: Large sliding inguino-scrotal hernia of the urinary bladder. A Case report and literature review	2018	59	M	Bilateral inguinal hernias with symptoms of frequent urination, difficulty emptying bladder	U/S: dilatation of the upper and middle sections of left ureter, slight uronephrosis of left kidney Urinary flow rate testing: reduced bladder capacity, severe bladder outlet obstruction CT/MRI: protrusion of the urinary bladder through left internal inguinal ring with extension into scrotum	Open preperitoneal inguinal herniaorrhaphy	No	None	
Coetzee *et al.*: Simple repair of giant inguinoscrotal hernia	2011	46	M	Left-sided inguinal hernia for past 2 years with main complaint of difficulty walking. No abdominal, GI or GU symptoms	Intraoperative hernia sac contents: most of the small bowel, cecum, appendix, ascending and transverse colon, omentum	Left inguinal transverse incision	No	None	Lichtenstein’s tension free
Sahsamanis at al: Treatment of a half century year old giant inguinoscrotal hernia. A case report	2016	77	M	Right-sided inguinal hernia for past 55 years with symptoms of increasing abdominal pain, dyspeptic symptoms, recurrent UTI	CT: Inguinosacral hernia containing the whole of the small bowel along with its mesentery	Right inguinal transverse incision connected to inguinal incision Extended lower midline incision	No	Right orchidectomy	Double mesh sutured on posterior wall of inguinal canal and posterior wall of rectus muscle
Staubitz *et al.*: Surgical treatment strategies for giant inguinoscrotal hernia- a case report with review of the literature	2017	63	M	Right-sided inguinal hernia over the past 10 years without groin pain ordigestion irregularities	CT: two-thirds of the small bowel, parts of ascending and transverse colon present in hernia sac	Combined open transabdominal and inguinal approach	Yes	Omentectomy, Right orchidectomy	Lichtenstein’s tension free
Tarchouli *et al.*: Giant inguinoscrotal hernia containing intestinal segments and urinary bladder successfully repaired by simple hernioplasty technique: a case report	2015	65	M	Right-sided inguinal hernia for past 12 years with symptoms of episodic abdominal pain, difficulty urinating, difficulty walking.	CT: voluminous hernia sac containing small and large bowel loops, greater omentum, urinary bladder	Right inguinal transverse incision	No	None	Lichtenstein’s tension free
Tahir *et al.*: Giant inguinoscrotal hernia: Case report and management principles	2008	50	M	Left-sided inguinal hernia for past 6 years without GI or GU symptoms	Intraoperative hernia sac contents: small bowel with its mesentery, appendix, cecum, ascending colon	Combined open transabdominal and inguinal approach	No	None	Lichtenstein’s tension free
Farshid *et al.*: Giant inguinoscrotal hernia repaired by combined Bassini and Lichtenstein technique	2019	68	M	Left inguinal hernia for past 20 years with symptoms of inguinal pain, difficulty walking,. No GI or GU symptoms	Intraoperative hernia sac contents: small bowel with mesentery, descending colon, greater omentum, urinary bladder	Left inguinal transverse incision	Yes	Partial omentectomy	Combined Bassini and Lichtenstein’s tension free
Bjurlin *et al.*: Clinical and radiographic findings of a sliding inguinoscrotal hernia containing the urinary bladder	2010	67	M	Right inguinal hernia with symptoms of difficulty urinating	CT: large right inguinal sliding hernia of the urinary bladder and omentum	Right inguinal transverse incision	Yes	Partial omentectomy	Lichtenstein’s tension free

## DISCUSSION

A comprehensive literature review of giant inguinal hernia cases was performed to demonstrate the various surgical approaches to managing this disease (summarized in Table 1). A total of 11 cases from 2008 to 2019 were used. Every patient in the literature review was a male with an average age of 62.3 years, ranging from 46–77 years old. Patient presentation ranged from asymptomatic to difficulty urinating to partial bowel obstruction. Majority of cases (7/11, 63.6%) only required a standard inguinal transverse incision with the remaining required addition of an open transabdominal approach. Roughly half of the cases required debulking to aid in reduction of the hernia. By far, the most common hernia repair technique was Lichtenstein’s tension free repair [1–11].

The preceding case of giant inguinal hernia repair provides a surgical approach with several unique features. First, a general surgeon and an urologist collaborated to treat this patient before, during and after surgery. Second, peritoneal fluid (3.9 L) was aspirated from the hernia sac to facilitate manual reduction of the hernia contents. Third, a total extraperitoneal abdominal incision was made to aid in reduction the hernia into the abdominal cavity.

## CONCLUSION

Surgical repair of giant inguinal hernias can be challenging and is associated with a variety of complications. Various modalities have been described to assist in hernia reduction including debulking, or, as in this case, aspiration of the hernia sac and a preperitoneal incision. Although Lichtenstein’s tension free repair seemed to be the most common technique (utilized in this case), no single treatment has been adopted as the standard approach to managing this uncommon presentation.

## CONFLICT OF INTEREST STATEMENT

None declared.

## FUNDING

None.
